# The Environmental Determinants of Skin Health: Linking Climate Change, Air Pollution, and the Dermatologic Disease Burden

**DOI:** 10.3390/ijerph22121820

**Published:** 2025-12-04

**Authors:** Tarek Zieneldien, Sophia Ma, Isabella J. Tan, Janice Kim, Daniel Busot, Bernard A. Cohen

**Affiliations:** 1 School of Medicine, Johns Hopkins University, Baltimore, MD 21205, USA; 2 Robert Wood Johnson Medical School, Rutgers, The State University of New Jersey, Piscataway, NJ 08901, USA; 3 College of Osteopathic Medicine, Michigan State University, East Lansing, MI 48824, USA; 4 College of Public Health, University of South Florida, Tampa, FL 33612, USA; 5 Department of Dermatology, The Johns Hopkins Hospital, Baltimore, MD 21287, USA

**Keywords:** air pollution, climate change, environmental stressors, dermatologic disorders

## Abstract

Climate change has a widespread impact on health across the continuum, influencing skin disease patterns, access to dermatologic care, and the burden of climate-sensitive conditions. Environmental changes driven by climate change impact the skin’s ability to maintain homeostasis, contributing to the onset and exacerbation of various dermatologic diseases. Psoriasis, acne vulgaris, atopic dermatitis, photoaging, melasma, and skin cancers have been associated with repeated exposure to rising levels of pollutants. Furthermore, the depletion of the stratospheric ozone layer has contributed to an elevated risk of developing skin cancer—including melanoma, basal cell carcinoma, and squamous cell carcinoma—due to increased exposure to ultraviolet radiation. Notably, while melanoma is linked to intense, intermittent UV exposure and sunburns, basal cell and squamous cell carcinomas are more strongly associated with cumulative or chronic sun exposure over a lifetime. According to the World Health Organization, air pollution contributes to more than 700,000 premature deaths each year, and a 1% decrease in ozone thickness corresponds with a 2% rise in melanoma incidence. This review also identifies research gaps, such as limited longitudinal studies, underrepresentation of pediatric and elderly populations, and limited exploration of nitrosative stress mechanisms. Due to these factors, practitioners should be aware of both the current and projected impacts of climate change in their regions to effectively identify and manage associated conditions and exacerbations.

## 1. Introduction

The skin is the largest organ in the body, covering its entire external surface and it serves as a primary barrier against environmental insults [1]. In recent years, the rising prevalence of skin disorders has paralleled growing concern over global environmental changes, specifically air pollution and climate change [2]. These issues transcend national boundaries, affecting both industrialized and developing regions through shifts in temperature, ultraviolet (UV) exposure, and urban air quality. For instance, elevated particulate matter exposure in Seoul, South Korea, has been associated with increased flares of atopic dermatitis, while similar correlations have been reported between pollution and pigmentation disorders in urban centers such as Beijing and Mexico City [3,4,5]. Likewise, studies in Europe and North America have demonstrated that chronic exposure to nitrogen dioxide and ozone contributes to inflammatory dermatoses [6,7]. These interrelated issues have emerged as pressing public health issues with dermatological implications. While air pollution has been established as a systemic health hazard affecting many systems, increasing morbidity and mortality [8], its effects on cutaneous health are increasingly recognized, but still underrepresented in clinical practice and research. Additionally, manifestations of climate change, including rising temperatures, increased ultraviolet (UV) radiation, altered humidity levels and more frequent weather events, are disrupting ecological balances and human health [2,9]. These environmental stressors may exacerbate existing dermatological conditions, by compounding oxidative stress, impairing skin barrier function, and provoking inflammatory cascades through complex interactions between pollution and climate shifts. This review aims to explore current literature regarding the effects of climate and air pollution on skin, elucidate underlying pathophysiological mechanisms, and highlight vulnerable groups. It will accomplish this by (1) summarizing major pollutants, climate stressors, and extreme weather events, (2) explaining key pathophysiological mechanisms, (3) reviewing dermatological disorders linked to these stressors, (4) discussing vulnerable populations and disparities, and (5) proposing clinical, public health, and research priorities. By emphasizing the significance of these evolving challenges, we aim to promote proactive strategies and promote interdisciplinary collaboration to protect skin health in a changing world.

## 2. Methodology

This narrative review was conducted to synthesize and critically evaluate current evidence on the dermatologic impacts of air pollution and climate change. Literature searches were performed across PubMed, Scopus, and Web of Science from database May 1975 through June 2025 using combinations of key terms including “*air pollution*,” “*particulate matter*,” “*ozone*,” “*polycyclic aromatic hydrocarbons*,” “*climate change*,” “*ultraviolet radiation*,” “*temperature*,” “*humidity*,” and “*skin*” or *“dermatologic disease*.” Boolean operators and Medical Subject Headings (MeSH) were used to refine results. Reference lists of relevant primary studies and review articles were also screened to identify additional sources. Studies were prioritized for inclusion if they: (a) reported original data on humans or used in vitro/in vivo models relevant to cutaneous health outcomes, (b) provided epidemiologic, mechanistic, or clinical evidence linking ambient air pollutants or climate-associated variables to skin physiology or dermatologic conditions, or (c) examined pollutants of contemporary concern (e.g., PM_2.5_, PM_10_, PAHs, ozone, nitrogen oxides, volatile organic compounds) or climate stressors (e.g., UV radiation, heat, humidity). Both observational and interventional designs were considered, along with key in vitro and in vivo mechanistic studies providing biologic plausibility. Additional emphasis was placed on high-quality studies assessing carcinogenic potential and oxidative pathways associated with fine particulate exposure to address emerging evidence linking PM and skin cancer risk.

Articles were excluded if they (a) focused exclusively on systemic disease outcomes without dermatologic endpoints, (b) examined occupational or indoor exposures not representative of ambient pollution, or (c) lacked clear exposure or outcome definitions.

Given the heterogeneity of exposure metrics and outcome definitions, a formal meta-analysis was not feasible, which represents a limitation as quantitative synthesis (such as pooled effect estimates) could not be performed. Instead, evidence was qualitatively synthesized and organized into five major domains: (1) characterization of air pollutants and climate stressors, (2) mechanistic pathways linking environmental exposures to cutaneous injury, (3) dermatologic conditions associated with these exposures, including recent evidence implicating PM in skin carcinogenesis, (4) vulnerable populations and global disparities, and (5) clinical, public health, and research implications. Studies with precise exposure characterization, validated dermatologic endpoints (e.g., EASI, PASI, DLQI), and contemporary relevance (recent epidemiologic or mechanistic advances) were prioritized to enhance comprehensiveness and reproducibility. Studies were appraised based on methodological design, sample size, and translational relevance, with greater weight given to controlled human exposure studies and longitudinal epidemiologic analyses. Mechanistic or in vitro investigations were interpreted as providing supportive biologic plausibility rather than direct clinical evidence.

Of the 108 references reviewed, 39 (36.11%) were primary research articles, predominantly observational studies (66.67%), with smaller proportions of experimental laboratory (23.08%), interventional (7.69%), and case report (2.56%) designs, while the remaining 69 (63.89%) were review or position papers.

## 3. Overview of Air Pollutants and Climate Stressors

### 3.1. Common Air Pollutants

Air pollution is a complex mixture of gaseous and particulate matter that includes polycyclic aromatic hydrocarbons, nitrogen dioxide, ozone, sulfur dioxide, tobacco smoke, and indoor air pollution (Table 1). [10]. The World Health Organization considers atmosphere (outdoor) and household (indoor) air pollution to be the greatest environmental health risk, causing more than 700,000 premature deaths each year [11]. Particulate matter (PM) is a type of air pollution that is composed of extremely small particles and liquid droplets that contain acids, organic chemicals, metals, and soil or dust particles [12]. Polycyclic aromatic hydrocarbons (PAHs) are lipophilic compounds that are byproducts of incomplete combustion of organic matter such as fossil fuels, tobacco smoke, and grilled foods [13]. Human exposure of PAHs can occur through different routes such as inhalation, dermal touch, and ingestion [14]. Inhalation is the dominant pathway, with urinary 3-hydroxybenzo[a]pyrene levels rising in proportion to ambient air PAH concentrations, reflecting real-time intake [15]. Skin also represents a significant route; for instance, firefighters’ skin PAH levels can exceed those in the surrounding air, underscoring cutaneous uptake as a major exposure pathway [16]. Eating contaminated food and drinking polluted water significantly contributes to exposure, with incremental lifetime cancer risks from local fish and water measured at 0.010 × 10^−3^ (i.e., 1 in 100,000) and 0.345 × 10^−3^ (i.e., 3.5 in 10,000), respectively [17]. Once PAHs adhere to the skin, they induce aryl hydrocarbon receptor (AhR) activation and reactive oxygen species generation which triggers pro-inflammatory cascades [18]. Nitrogen dioxide is an ambient air pollutant that is a traffic-related pollutant [19]. Increased levels of exposure to nitrogen dioxide contributes to oxidative stress and barrier dysfunction leading to skin surface changes such as the formation of lentigines [20]. Tropospheric ozone, a harmful secondary air pollutant, is generated at ground level through photochemical reactions between sunlight, nitrogen oxides, and volatile organic compounds—distinct from stratospheric ozone, which forms the protective “ozone layer” [21]. Sulfur dioxide is a byproduct of coal and oil combustion, mainly from industrial manufacturing [22]. It is a known respiratory and mucocutaneous irritant and may trigger eczema-like eruptions and contact dermatitis, particularly in individuals with sensitive skin or existing dermatoses [23,24]. Furthermore, cigarette smoke contains over 4000 chemicals, including PAHs, heavy metals, and formaldehyde which accelerate collagen degradation and promote wrinkle formation, particularly periorbital and perioral aging [25,26].

### 3.2. Climate Related Stressors

The skin’s direct exposure to climate elements such as temperature, humidity, and solar radiation makes it highly susceptible to these evolving stressors. With increased UV radiation due to ozone layer depletion, it leads to enhanced UV exposure, a major driver of DNA damage and photoaging [41]. Increased UV exposure is a significant driver in skin carcinogenesis including basal cell carcinoma, squamous cell carcinoma, and melanoma [42]. Elevated ambient temperatures and more frequent heatwaves can increase sweat gland activity and vasodilation which can aggravate inflammatory dermatoses such as atopic dermatitis [43,44]. Additionally, fluctuations in humidity, both high and low, affect skin homeostasis. In low humidity environments it can lead to increased transepidermal water loss, skin dryness, and barrier dysfunction worsening conditions such as atopic dermatitis and psoriasis [45,46]. In high humidity environments, increased moisture can lead to higher sweat and oil production, fostering microbial growth and elevating the risk of skin irritation, infection, and flare-ups of conditions such as eczema [47]. Geographic areas with elevated humidity are associated with poorly controlled eczema, likely due to increased sweating and a favorable environment for irritants and microbes to affect the skin barrier [48]. Moreover, climate change has intensified the frequency and severity of natural disasters such as flooding and wildfires which can trigger dermatological conditions and infections. Flooding significantly increases the incidence of dermatological conditions due to trauma, prolonged water exposure, poor sanitation, and contamination with sewage and pollutants [49]. Common post-flood skin issues include traumatic wounds, bacterial and fungal infections, and inflammatory dermatoses. Wildfire smoke contains harmful pollutants such as PM, carbon monoxide, sulfur dioxide, nitrogen dioxide, volatile organic compounds (VOCs), and ozone, which can negatively affect skin health through direct penetration, follicular absorption, or systemic circulation [50]. These pollutants disrupt the skin barrier by downregulating key structural proteins like filaggrin and loricrin, increasing transepidermal water loss and weakening defense against external insults [51]. These extreme weather events significantly impact healthcare delivery, as damage often occurs to essential infrastructure such as power grids, hospitals, water sanitation facilities, and cold-chain storage required for vaccines and medications [49]. Furthermore, emergency services are also typically disrupted when roads are flooded, blocked by debris, or damaged [49]. Homes and personal property may also be damaged, leading to displacement, as well as the loss of transportation means and critical medications. This, in turn, leads to significant emotional stress, which may exacerbate dermatologic and other comorbid conditions [52]. 

### 3.3. Pathophysiological Mechanisms

**Figure 1:**
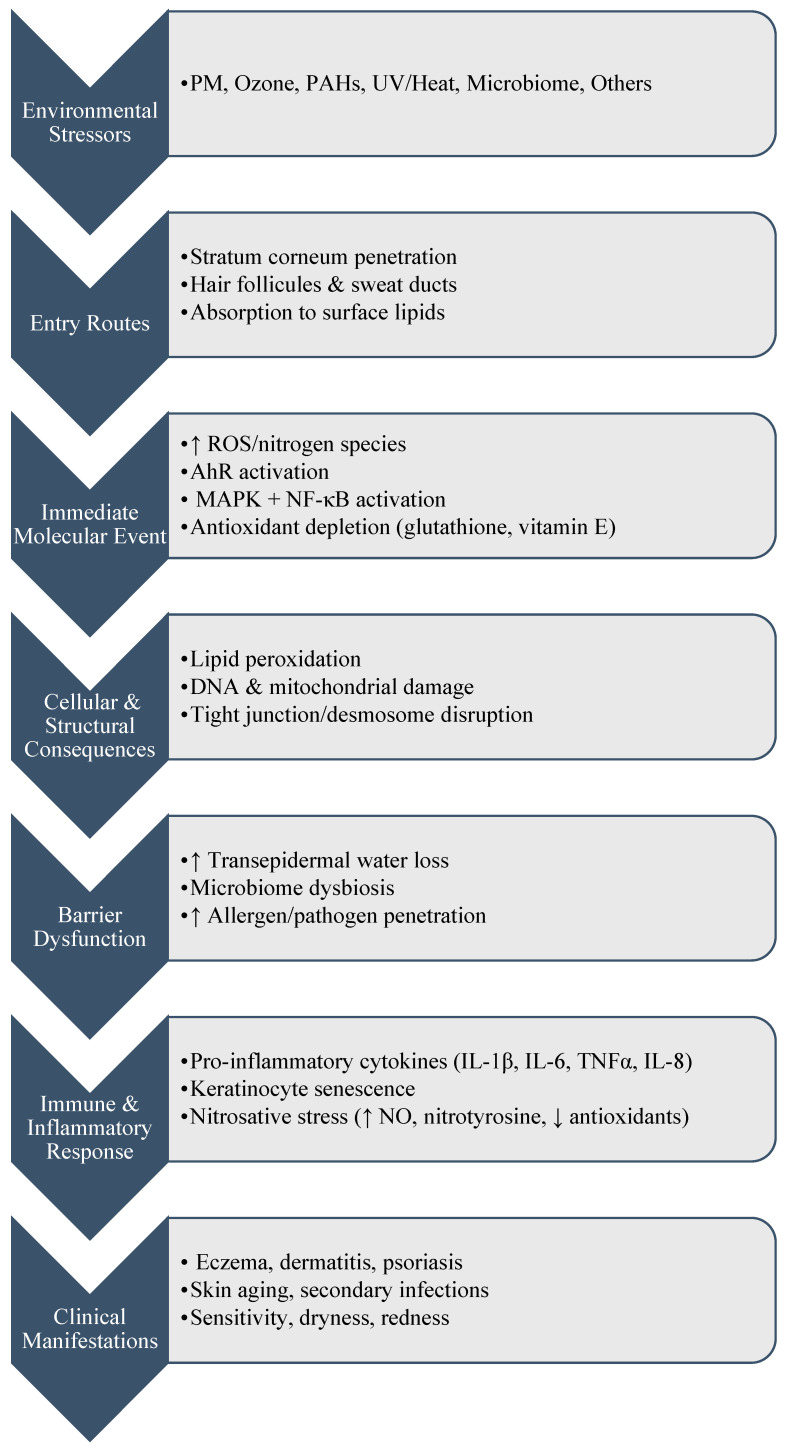
Mechanistic Pathways Linking Environmental Stressors to Cutaneous Barrier Dysfunction and Inflammatory Skin Disease.

The skin’s response to environmental stressors involves a complex relationship between barrier function, immune surveillance, and structural integrity. The pathophysiological mechanisms linking climate change to skin pathology involve excess reactive oxygen species (ROS) generation, oxidative stress, pro-inflammatory signaling, barrier dysfunction with increased transepidermal water loss, and microbiome dysbiosis (Figure 1) [53,54,55]. Air pollutants such as particulate matter (PM), ozone, and polycyclic aromatic hydrocarbons (PAHs) access the skin by diffusing through the stratum corneum, penetrating hair follicles and sweat ducts, or adsorbing to surface lipids [32]. Ultrafine PM (<0.1 µm) can translocate into deeper epidermal layers, while ozone reacts with squalene and other surface lipids to generate secondary ozonides and aldehydes that propagate ROS beyond the stratum corneum [32]. Pollutant-derived ROS activate the AhR and stimulate NF-κB and MAPK pathways, amplifying pro-inflammatory cytokine and chemokine release [32]. In parallel, climate-related heat and UV exposure deplete antioxidants such as glutathione and vitamin E, reducing cutaneous redox defenses and compounding oxidative stress [56]. Sustained ROS overload leads to lipid peroxidation, mitochondrial dysfunction, DNA damage, telomere shortening, and impaired autophagy, which collectively drive keratinocyte senescence, hallmarks of extrinsic skin aging and inflammatory dermatoses [57]. In addition, patients with cutaneous lichen planus demonstrate elevated serum levels of nitrite, nitrate, and symmetric dimethylarginine, alongside reduced total antioxidant status, highlighting nitrosative stress as a parallel pathway linking environmental stressors to inflammation and impaired antioxidant defense [58]. In patients with vitiligo, markers of nitrosative stress including nitric oxide, nitrotyrosine (3 NTx), and Raftlin were significantly elevated, while antioxidant enzyme activities including catalase, superoxide dismutase, glutathione peroxidase, and glutathione S transferase were reduced, indicating that nitrosative stress plays a central role in melanocyte damage and disease progression [59]. Additionally, with the activation of pro-inflammatory transcription factors particularly NF-κB and MAPK, it leads to release of cytokines such as IL-1β, IL-6, TNF-α, and IL-8, which contribute to skin redness, edema, and the worsening of conditions like atopic dermatitis and psoriasis [60]. Moreover, exposure to airborne pollutants can lead to transepidermal water loss and impair water retention, resulting in dryness, flaking, and heightened skin sensitivity [61]. UVB radiation exacerbates this process by impairing the expression and function of tight junction proteins (e.g., claudins and occludins) and degrading desmosome components that maintain cell–cell adhesion [62]. The cumulative effect is a weakened epidermal barrier that is more permeable to allergens, pathogens, and irritants, which facilitate cutaneous inflammation and increase the risk for eczematous dermatitis (including atopic and contact dermatitis), and secondary infections.

Emerging evidence indicates that chronic exposure to fine particulate matter (PM_2.5_, PM_10_) can induce carcinogenic changes in the skin through various mechanisms. PM may penetrate the epidermis, leading to oxidative DNA damage, lipid peroxidation, and sustained inflammatory signaling [11]. Key mechanisms implicated in PM-driven skin carcinogenesis include increases ROS generation, DNA strand breaks, and activation of the AhR, which modulates cell proliferation and apoptosis pathways [11]. Even more, PAHs adsorbed on PM can enhance these effects by promoting genotoxicity and mutagenesis [63]. Recent mechanistic studies highlight that persistent AhR activation by PM-bound ligands disrupts cell cycle regulation and impairs DNA repair mechanisms, thereby facilitating carcinogenic transformation of keratinocytes. Additionally, PM exposure has been associated with epigenetic alterations and telomere shortening, further contributing to genomic instability in skin cells [64].

### 3.4. Dermatological Disorders Linked to Pollution and Climate Change

Environmental exposures such as air pollution and climate change are increasingly recognized as aggravating factors in a range of dermatological disorders such as atopic dermatitis, acne, psoriasis, urticaria, skin cancer, rosacea, and pigmentary conditions (Table 2) [55]. Atopic dermatitis (AD) is strongly influenced by environmental conditions as urbanization, high levels of PM and ozone, and low humidity environments all correlate with AD prevalence and severity [32]. Airborne particles degrade the skin barrier and increase IL-4/IL13 mediated inflammation and may also alter the cutaneous microbiome, further aggravating AD [65]. In a study conducted in Seoul, South Korea, investigators found that children 5 years or younger living in highly polluted cities had significantly higher rates of AD flares [4]. Additionally, pollutants such as PAHs, nitric oxide (NO), and cigarette smoke may induce sebocyte hyperactivation, increased sebum oxidation and comedogenesis [50]. In China, there was a study conducted that found increased cutaneous exposure to PMs was significantly associated with a higher number of lesions and hyperseborrhea [3]. While the exact mechanism of acne formation and air pollutants is unknown, it could be due to air pollution inducing oxidative stress in the skin that causes lipid peroxidation and oxidative DNA damage [66]. Evidence indicates that pollution-induced oxidative stress contributes to cutaneous abnormalities, with diesel exhaust particles shown to drive melanogenesis in human skin via oxidative pathways [5]. Moreover, psoriasis is an immune-mediated skin disorder with systemic inflammation in which Th17 cells and IL-17 play a crucial role in its pathogenesis [67]. The activation of aryl hydrocarbon receptors (AhR) receptors by air pollutants has been proposed as a potential pathogenic mechanism in the development of psoriasis [68]. A study conducted in Verona, Italy found a correlation between air pollution exposure and increased psoriasis activity, along with a higher likelihood of flares [69]. Also, global warming and pollutant-driven changes in aeroallergens have contributed to increased cases of contact dermatitis, allergic urticaria, and photosensitivity [70]. Longer allergen season and increased plant volatile organic compounds have contributed to the sensitization as these allergens may act with pollutants to enhance mast cell degranulation and IgE responses [71,72]. Furthermore, a correlation between UV radiation and pollutants in amplifying skin cancer incidence has been suggested [73]. A few studies, primarily based on animal models, have indicated that particulate matter (PM) and polycyclic aromatic hydrocarbons (PAHs) may significantly contribute to skin carcinogenesis [73]. Recent studies have provided growing evidence for the association between fine particulate matter (PM_2.5_) and the risk of skin cancer, including melanoma [74,75,76,77]. Additionally, a 1% decrease in the ozone thickness corresponds with a 2% rise in UVB irradiation-associated melanoma and a 3–4% increase in squamous cell carcinoma [78]. Lastly, the activation of AhR/MAPK signaling in melanocytes in response to air pollutants such as PAH or dioxins promotes the proliferation of melanocytes, resulting in hyperpigmentation and melasma [41,66]. Several epidemiological studies suggest a link between chronic exposure to particulate matter and increased risk of non-melanoma skin cancer, likely through oxidative stress, mutagenic DNA damage, and persistent activation of pathways including the AhR [79]. Although the association is stronger for non-melanoma skin cancers (basal cell carcinoma, squamous cell carcinoma), ongoing research is examining the relationship between PM exposure and melanoma as well. Cohort data from urban populations have reported a positive correlation between PM_2.5_ levels and skin cancer incidence, emphasizing the need for public health mitigation and further study [79]. As the global environment shifts continue to accelerate, it reinforces the significant and multifaceted role environmental factors play in the onset and exacerbation of skin conditions.

## 4. Vulnerable Populations and Global Disparities

Climate change and air pollution disproportionately burden vulnerable groups, exacerbating skin disorders in children, outdoor workers, the elderly, and socioeconomically disadvantaged communities, particularly in low- and middle-income countries (LMICs), despite these countries contributing less to the climate crisis than high-income countries [52]. The United States, China, and the European Union, which are leading greenhouse gas emitters, contribute approximately 41.5% of total global emissions, whereas the 100 countries with the lowest emissions combined are responsible for just 3.6% [86]. 

Children are particularly susceptible due to their developing immune systems and skin barrier, which increase absorption of airborne pollutants and exacerbate conditions such as atopic dermatitis. Studies have shown that short-term exposure to ambient air pollution in cities such as Chongqing, China, which experiences high levels of air pollution, may be associated with an increase in outpatient visits for childhood atopic dermatitis [87]. The elderly also face heightened vulnerability, since physiological aging, immunological decline, and cumulative environmental exposures compromise skin barrier function, leading to increased incidence and severity of inflammatory skin diseases, infections, and malignancies, especially during periods of poor air quality or extreme weather events [88,89,90]. Aging-related skin changes, such as decreased skin hydration, altered pH of the stratum corneum, increased transepidermal water loss, and heightened cytokine-mediated inflammation, result in skin that is more prone to environmental insults, including air pollutants, extreme temperatures, and ultraviolet radiation [90,91].

Occupational exposure further amplifies risk among outdoor workers, such as construction and agriculture laborers, who are in frequent contact with ultraviolet radiation, temperature extremes, and ambient pollutants. This leads them to be susceptible to higher rates of contact dermatitis, environmentally triggered dermatoses, photodamage, and skin cancer [92]. Furthermore, socioeconomic disparities play a critical role, with lower income and limited health literacy being consistently associated with more severe and persistent atopic dermatitis, lower housing quality has been associated with the development of rosacea, longer time to treatments is often seen in skin cancer among low SES patients, and greater exposure to environmental hazards [93]. In contrast, high SES individuals often have resources that protect them from elevated exposures to hazards, such as indoor work environments, access to private transportation, living in newer buildings, and often, the ability for climate control and filtration in indoor environments [94]. Higher prevalence of atopic dermatitis in affluent populations highlights a counterintuitive risk and suggests underdiagnosis in lower SES groups may mask the true burden of disease [95].

## 5. Clinical and Public Health Implications

Dermatologists play a crucial role in recognizing and diagnosing skin conditions that may be triggered or exacerbated by environmental exposures. This expertise is important as shifts in climate and air quality have altered the epidemiology and severity of conditions like atopic dermatitis, psoriasis, scabies, pyoderma, and pigmentary disorders [96]. Beyond their role in diagnosis and management, dermatologists also have the duty to counsel on protective measures against environmental insults. This includes recommending sun protective clothing such as swim shirts, sunglasses, and hats, topical broad-spectrum sunscreens, and barrier creams [97]. Furthermore, patient education on lifestyle modifications also plays an important role, with knowledge about avoiding outdoor activities during heat waves or high pollution periods being integral to comprehensive care.

Given the growing body of evidence linking environmental change to dermatologic disease, there is an urgent need for dermatologists to engage in policy advocacy and collaborate with climate scientists and public health experts. Such interdisciplinary efforts are vital to inform regulatory standards, promote sustainable urban planning, and develop community-level interventions that minimize exposure to harmful environmental factors [2]. As of now, rising average temperatures and changing wind patterns have significantly contributed to the expansion of the geographic distribution and incidence of fungal skin diseases, such as coccidioidomycosis. The incidence of infectious skin disease has been shifting due to climate conditions that favor the transmission of microbes, and global skin cancer risk is rising due to ozone layer depletion [98,99,100,101]. Dermatologists also have a social responsibility to promote sustainable practices in the clinic through water conservation efforts, minimizing regulated medical waste, utilizing nontoxic cleaning products, and participating in the purchasing of greener products [102]. By switching to products that have less packaging that is free of di-ethylhexyl phthalate, polyvinyl chloride, and latex, allergic reactions can potentially be prevented, while also decreasing burns and asthma exacerbations [102]. 

## 6. Future Directions and Research Gaps

While recognition of the dermatological consequences of environmental changes is growing, significant gaps in clinical and scientific understanding persist, underscoring the need to address these gaps to develop effective prevention, diagnosis, and treatment strategies [103]. Until the ozone layer fully recovers, projected by 2040, elevated UV exposure continues to drive dermatologic conditions such as skin cancer, photoaging, and pigmentary disorders. Concurrently, other environmental stressors, including air pollution and climate-related exposures, remain significant threats to skin health, underscoring the need for continued research, public education, and targeted prevention strategies [104]. While studies exist on the relationship between environmental pollution and skin disorders, they are mostly limited to urban centers, highlighting the need for longitudinal cohort studies across diverse geographic and socio-economic populations to establish causality, quantify exposure-response relationships, and assess temporal trends. These complex skin conditions require coordinated, multisystem care and healthcare professionals play a vital role not only in clinical management but also in advancing community education, institutional response, and advocacy through their health systems and professional networks [52]. By emphasizing the health impacts of climate change, they have a powerful opportunity to educate the public and policymakers to promote evidence-based solutions and advocate for interventions that protect human health, as referenced by the American Academy of Dermatologists Statement on Climate and Health declaring a commitment to “raise awareness about the effects of climate change on skin health and skin disorders” [105]. Moreover, further studies are needed to understand how environmental exposures differentially impact pediatric, geriatric, ethnic minority, and immunocompromised populations. These studies should incorporate comprehensive molecular profiling (genetic, epigenetic, and metabolite analyses) alongside detailed environmental exposure assessments and account for socio-environmental and occupational factors that influence susceptibility [32]. 

Future research should also prioritize longitudinal, prospective cohort designs integrating personal exposure monitoring and geospatial analyses to refine dose–response relationships. Multi-omics approaches combining genomic, epigenomic, and metabolic phenotyping with clinical data are needed to elucidate mechanistic pathways and identify biomarkers of susceptibility. Interdisciplinary collaboration between dermatology, environmental science, epidemiology, public health, and policy sectors must be fostered, leveraging partnerships between academic institutions, government agencies, industry, and community organizations. Funding opportunities exist through agencies such as the National Institute of Environmental Health Sciences, the CDC Climate and Health Program, the EPA STAR Program, and international funders like the Wellcome Trust, which should be actively pursued and continued to be supported. Dermatologic societies, including the American Academy of Dermatology, European Academy of Dermatology and Veneeology, among others have a vital role in leading educational efforts through official policy statements, research prioritization, sustainable practice initiatives, and policy advocacy [106,107]. These are important considering that a unique aspect of the dermatologic field encompasses the considerable use of topical formulations, such as sunscreens, medical ointments, and cosmeceuticals, which may introduce active compounds, synthetic polymers, and other poorly biodegradable ingredients into waste streams [107]. Combined with petroleum-derived bases and small plastic packaging for samples which may have an environmental footprint out of proportion to the product volume, these products contribute to a significant ecological burden [108]. Ultimately, bridging these research and clinical gaps is essential for developing effective prevention, diagnosis, and treatment strategies for environmental skin diseases.

## 7. Conclusions

Air pollution and climate change represent significant and growing challenges to dermatological health. This review has outlined environmental stressors including particulate matter, ultraviolet radiation, temperature extremes, and shifting humidity patterns as increasingly implicated in the pathogenesis and exacerbation of a wide range of skin conditions. These exposures act through complex biological mechanisms such as oxidative stress, inflammation, skin barrier disruption, and immune dysregulation, contributing to dermatological conditions. Despite a growing body of literature, a gap remains, particularly regarding the long-term exposure effects, population specific vulnerabilities, and molecular mechanisms regarding the changing environmental conditions and the effects on skin. Addressing these gaps requires coordinated effects across clinical, research, and public health sectors. As climate change and pollution continue to threaten skin health globally, urgent, coordinated efforts across research, clinical practice, and policy domains are needed. Adopting standardized methodologies, investing in large-scale, diverse cohorts, and developing clinical guidelines that incorporate environmental risk factors will advance care and prevention strategies. Dermatologists, supported by leading dermatologic societies, must lead in policy advocacy, sustainable practice adoption, and interdisciplinary collaboration to mitigate the rising burden of environmental skin disease, safeguard vulnerable populations, and promote health equity.

## Figures and Tables

**Table 1:** Overview of the Mechanisms and Effects of Novel Pollutants.

Stressor	Description	Mechanism	Associated Effect
**Micro- and nanoplastics (MNPs) [27,28]**	Plastic fragments from environmental degradation of larger plastics or direct industrial/consumer sources; microplastics (<5 mm) and nanoplastics (<1 μm) detected in air, water, food, and human tissues.	Exposure via inhalation, dermal contact, or ingestion; MNPs generate reactive oxygen species (ROS), disrupt epidermal barrier lipids, provoke keratinocyte inflammatory signaling (IL-1, IL-6, TNF), and perturb the skin microbiome. Nanoscales may translocate across compromised epithelia.	Mechanistic evidence suggests potential to impair barrier function and drive cutaneous inflammation, raising concern for contributions to premature skin aging and dermatitis, though direct human evidence remains limited.
**Per- and polyfluoroalkyl substances (PFAS) [29,30,31]**	Synthetic fluorinated compounds used for water-, grease-, and stain-resistant properties in textiles, packaging, cookware, and cosmetics; persistent in environment and bioaccumulative in humans.	Dermal contact and ingestion from consumer products; PFAS disrupt epidermal lipid metabolism and skin barrier composition, induce oxidative stress, dysregulate cytokines, and modulate systemic immune and epigenetic programs.	Associated with increased risk or worsening of immune-mediated dermatoses (e.g., atopic dermatitis, psoriasis), impaired barrier repair, altered skin lipid composition, and chronic pruritus/eczema-type presentations.
**Ultrafine particles/traffic-related PM [32,33,34]**	Airborne particles < 100 nm from combustion (traffic, industrial emissions, indoor fuel burning); capable of deep lung and skin deposition.	Dermal deposition and inhalation; activates AhR, oxidative pathways (ROS), lipid peroxidation, MAPK/NF-κB signaling, and downstream pro-inflammatory and matrix remodeling programs in keratinocytes and melanocytes.	Drives pigmentary disorders and hyperpigmentation (AhR-mediated melanogenesis), accelerates photoaging (MMP induction), exacerbates atopic dermatitis and acne via sebum oxidation and inflammatory cascades.
**Tire/road-wear particles (TRWP) [35,36,37]**	Particulate mixtures from mechanical abrasion of vehicle tires, brakes, and road surfaces; composed of rubber polymers, fillers, additives, metals; disperses into air, soil, and water.	Dermal and environmental exposure; TRWPs induce oxidative stress, inflammatory signaling, and perturb lipid metabolism in epidermal cells. Metal and PAH fractions (e.g., benzo[a]pyrene, naphthalene) contribute chemical-mediated toxicity.	Mechanistic data suggest potential for oxidative injury and low-grade chronic inflammation, possibly contributing to eczematous dermatitis (e.g., atopic or contact dermatitis), or accelerated extrinsic skin aging; human data remain sparse.
**Engineered nanoparticles (TiO_2_, ZnO, Ag, etc.) [38,39,40]**	Man-made nanomaterials intentionally produced for sunscreens, cosmetics, textiles, and electronics; metal and metal oxide nanoparticles with unique size- and surface-dependent properties.	Dermal exposure from topical products; can generate ROS, mitochondrial dysfunction, DNA/protein oxidative damage (effects amplified by UV co-exposure or compromised skin), modulated by particle coating, aggregation, and dose.	May increase epidermal cell stress and barrier impairment (↑TEWL), potentiate UV-induced damage, and for some metal NPs or poorly formulated products, contribute to contact sensitization or delayed healing in compromised skin.

**Table 2:** Clinical Evidence Linking Environmental Pollutants to Dermatologic Conditions.

Study	Population	Pollutant	Outcome	Findings	Additional Information
**Huss-Marp., 2006 [80]**	12 atopic eczema (atopic dermatitis), 12 healthy adults	Volatile organic compounds (VOCs) and Der p 1 (house dust mite allergen)	Trans epidermal water loss (TEWL), dermal blood flow, atopy patch test (APT) reactivity	VOCs significantly increased TEWL (+34%, 95% CI: 7–69%); in AE patients, VOCs caused increased dermal blood flow; 6/7 AE patients showed enhanced APT reactions post-VOC exposure	Short-term controlled human exposure study; mechanistic evidence for pollution-induced barrier dysfunction and allergen sensitivity
**Makino et al., 2018 [81]**	30 healthy adults	Air pollution/urban pollution mix (PM, PAHs etc.—study tested a topical day/night “LVS” product intended to protect vs. pollution)	Clinical skin aging/visible skin damage endpoints (wrinkles, redness, skin tone evenness, tactile roughness)	After 8 weeks in subjects living/working in a severely polluted environment (AQI reported > 300 during study window), treatment with the two-part skincare system (LVS) produced significant improvements vs. placebo in clinical grading (crow’s feet wrinkles, overall skin damage, tone evenness, roughness, visible redness). Biochemical markers (SQOOH and MDA) decreased on skin swabs and biopsies supported improved biomarkers.	Randomized, double-blind, placebo-controlled clinical usage study; endpoints included clinical grading, photography, sebum/skin swabs, biopsies.
**Bellinato et al., 2023 [82]**	169 patients included in analysis (from 528 eligible; data on 1130 follow-up visits and ~5840 pollutant measurements).	PM_10_, PM_2.5_, NO_2_, NO_x_ (ambient air pollutants measured from local monitoring station)	Atopic dermatitis (AD) flares (EASI score used—flare defined as EASI > 8)	Case-crossover analysis: short/medium-term increases in PM_10_, PM_2.5_, NO_2_ and NO_x_ were associated with higher odds of AD flare in patients with moderate-to-severe AD treated with dupilumab. Example: every 10 µg/m^3^ increase at 60 days was associated with 82% (PM_10_), 67% (PM_2.5_), 113% (NO_2_) increased odds of flare (time windows varied; dose–response shown).	Observational case-crossover of patients on dupilumab (each patient as their own control). Retrospective extraction from clinical records (December 2018–December 2021).
**Xiong et al., 2025 [83]**	451,064 UK Biobank participants (enrolled 2006–2010; followed to 2022); 4414 incident psoriasis cases during follow-up	Joint exposure score including PM_2.5_, PM_2.5–10_, PM_10_, NO_2_, NO_x_; also PM_2.5_ absorbance	Incident psoriasis (new diagnosis during follow-up)	Prospective cohort: higher combined air pollution exposure (air pollution score) associated with modestly increased risk of incident psoriasis overall; effect was stronger among those with high genetic risk (PRS). HRs for highest exposure/group with high genetic risk were elevated (e.g., HRs reported for various pollutants in high genetic risk + high exposure group up to ~1.7–1.8)	Large prospective epidemiologic study using UK Biobank exposures and polygenic risk score (PRS). Long median follow-up (~13.8 years). Good power; provides interaction with genetic susceptibility
**Stelmach et al., 2025 [84]**	147 participants from the Polish Mother and Child Cohort (prenatal maternal urine and child urine measurements; children age 2 assessed)	Phthalates (prenatal maternal urine metabolites: monobenzyl phthalate and others measured)	Early eczema/atopic dermatitis and food allergy at age 2	Higher maternal prenatal monobenzyl phthalate concentrations were associated with increased risk of food allergy in children in the first 2 years (OR 4.17, 95% CI 1.17–17.89). No clear associations were seen with child urine metabolites and allergic symptoms in this analysis.	Birth-cohort/exposure biomarker study (urine phthalate metabolites measured during 3rd trimester and in children at age 2). Observational; adjusted logistic regression used
**Stelmach et al., 2014 [56]**	501 children (Polish Mother and Child Cohort Study, birth cohort evaluated for early outcomes in first year)	Multiple environmental exposures; tobacco exposure (cotinine) and ambient traffic/PM_10_ exposure evaluated	Atopic dermatitis and early wheeze/food allergy in first year of life	Among multiple factors, maternal exposure to increased PM_10_ concentration had a positive association with atopic dermatitis in univariate analyses. Other predictors: parental atopy, paternal education, frequency of house cleaning; breastfeeding reduced risk. Study supports traffic/PM exposure as a possible early risk factor.	Inner-city birth cohort analyses; exposure assessment via questionnaire and cotinine; part of clinical trial registration NCT01861548. Observational; some associations attenuated after multivariate adjustment (authors report which associations persisted).
**Torii et al., 2011 [85]**	Human PBMC samples: 27 healthy volunteers (used to define normal Th17%), 33 psoriasis patients who were smokers, and 21 psoriasis patients who were non-smokers—(PBMC analysis described).	Tobacco smoke (cigarette smoking; tobacco smoke extract used in vitro)	Immunologic endpoint relevant to psoriasis (Th17 percentage among CD3^+^ cells; IL-17/IL-22 expression)	Smokers (psoriasis patients who smoke) had higher circulating Th17% vs. non-smoker patients and healthy volunteers. Tobacco smoke extract induced Th17 generation in vitro and increased IL-17 and IL-22 expression—providing a mechanistic link between smoking (a pollutant/exposure) and psoriasis-relevant immune activation.	Ex vivo human PBMC measurements combined with in vitro TSE (tobacco smoke extract) experiments; publication type labeled as “Letter” but contains human subject data and mechanistic experiments. Not an epidemiologic trial of disease incidence but supports mechanistic/clinical implications.

## Data Availability

No new data were created or analyzed in this study. Data sharing is not applicable to this article.

## Abbreviations

UV	Ultraviolet
PAH	Polycyclic aromatic hydrocarbons
PM	Particulate matter
VOCs	Volatile organic compounds
NF-κB	Nuclear Factor kappa-light-chain-enhancer of activated B cells
MAPK	Mitogen-Activated Protein Kinase
IL-1β	Interleukin-1 beta
IL-6	Interleukin-6
TNF-α	Tumor Necrosis Factor alpha
IL-8	Interleukin-8
IL-17	Interleukin-17
Th17	T helper 17 cell
AD	Atopic dermatitis
NO	Nitric oxide
AhR	Aryl hydrocarbon receptor
